# In Vivo Inhibition of miR-34a Modestly Limits Cardiac Enlargement and Fibrosis in a Mouse Model with Established Type 1 Diabetes-Induced Cardiomyopathy, but Does Not Improve Diastolic Function

**DOI:** 10.3390/cells11193117

**Published:** 2022-10-03

**Authors:** Bianca C. Bernardo, Gunes S. Yildiz, Helen Kiriazis, Claudia A. Harmawan, Celeste M. K. Tai, Rebecca H. Ritchie, Julie R. McMullen

**Affiliations:** 1 Baker Heart and Diabetes Institute, Melbourne, VIC 3004, Australia; 2 Department of Diabetes, Central Clinical School, Monash University, Clayton, VIC 3800, Australia; 3 Department of Paediatrics, University of Melbourne, Parkville, VIC 3010, Australia; 4 Baker Department of Cardiometabolic Health, University of Melbourne, Parkville, VIC 3010, Australia; 5 Monash Institute of Pharmaceutical Sciences, Monash University, Royal Parade, Parkville, VIC 3052, Australia; 6 Department of Physiology, Monash University, Clayton, VIC 3800, Australia; 7 Department of Physiology, Anatomy and Microbiology, La Trobe University, Melbourne, VIC 3086, Australia

**Keywords:** diabetes, microRNA, miR-34a, cardiomyopathy, fibrosis

## Abstract

MicroRNA 34a (miR-34a) is elevated in the heart in a setting of cardiac stress or pathology, and we previously reported that inhibition of miR-34a in vivo provided protection in a setting of pressure overload-induced pathological cardiac hypertrophy and dilated cardiomyopathy. Prior work had also shown that circulating or cardiac miR-34a was elevated in a setting of diabetes. However, the therapeutic potential of inhibiting miR-34a in vivo in the diabetic heart had not been assessed. In the current study, type 1 diabetes was induced in adult male mice with 5 daily injections of streptozotocin (STZ). At 8 weeks post-STZ, when mice had established type 1 diabetes and diastolic dysfunction, mice were administered locked nucleic acid (LNA)-antimiR-34a or saline-control with an eight-week follow-up. Cardiac function, cardiac morphology, cardiac fibrosis, capillary density and gene expression were assessed. Diabetic mice presented with high blood glucose, elevated liver and kidney weights, diastolic dysfunction, mild cardiac enlargement, cardiac fibrosis and reduced myocardial capillary density. miR-34a was elevated in the heart of diabetic mice in comparison to non-diabetic mice. Inhibition of miR-34a had no significant effect on diastolic function or atrial enlargement, but had a mild effect on preventing an elevation in cardiac enlargement, fibrosis and ventricular gene expression of B-type natriuretic peptide (BNP) and the anti-angiogenic miRNA (miR-92a). A miR-34a target, vinculin, was inversely correlated with miR-34a expression, but other miR-34a targets were unchanged. In summary, inhibition of miR-34a provided limited protection in a mouse model with established type 1 diabetes-induced cardiomyopathy and failed to improve diastolic function. Given diabetes represents a systemic disorder with numerous miRNAs dysregulated in the diabetic heart, as well as other organs, strategies targeting multiple miRNAs and/or earlier intervention is likely to be required.

## 1. Introduction

The number of people with diabetes has been rising globally and was estimated to exceed 450 million adults worldwide in 2017, representing approximately 8.4% of adults (18–99 years). By 2045, the prevalence is predicted to rise to 9.9%, representing over 690 million adults [1]. Diabetes is associated with increased mortality and morbidity, attributed largely to cardiovascular and kidney disease [2,3]. The heart undergoes adverse remodeling in a setting of diabetes and this is associated with an increased risk of heart failure and sudden death after myocardial infarction [4]. This elevated risk in diabetic patients remains even after adjusting for age, blood pressure, weight, cholesterol, and coronary artery disease [5,6,7]. The terms “diabetic cardiomyopathy” and “diabetic heart” have been used to define the cardiac dysfunction which can occur in diabetic patients in the absence of other factors including coronary artery disease and hypertension. Two key features of the diabetic heart include diastolic dysfunction and cardiac fibrosis [2,3,5,6]. There is no specific therapy for the diabetic heart, and with the exception of sodium-glucose contransporter-2 inhibitors (SGLT2i), therapies have not demonstrated positive cardiovascular outcomes (including thiazolidinediones, incretin-based therapies (glucagon-like peptide 1 and dipeptidyl peptidase-4 inhibitors)) [5,7,8,9]. There remains an unmet need to develop novel therapies for the diabetic heart.

MicroRNAs (miRNAs) are short strands of RNA that are not transcribed into protein, but instead, regulate the expression of many genes by interacting with specific sites in 3′ untranslated regions of messenger transcripts to prevent protein translation and gene expression [10]. miRNAs play a crucial role in health and disease, and as such, molecular tools to manipulate miRNA activity have been developed and proved successful in preclinical disease models [11,12]. Furthermore, some have entered clinical trials in patients with hepatitis C [13], cancer, type 2 diabetes, and non-alcoholic fatty liver diseases [14,15]. Alterations in miRNA expression in cardiac tissue from diabetic and non-diabetic mice have been identified from profiling studies [16,17], and represents a new class of targets for the development of therapeutics for diabetic cardiomyopathy.

Over the past decade, miRNA-34a has received considerable attention for its therapeutic potential in the heart. Expression of miR-34a is elevated in different animal models of cardiac stress [18,19,20,21,22,23], and was also shown to be elevated in the human failing heart [24,25]. We, and others, have demonstrated that therapeutic inhibition of miR-34a is beneficial in settings of heart pathology such as aging, pressure overload-induced pathological hypertrophy, acute myocardial infarction and dilated cardiomyopathy [18,19,20,21,22,23]. However, the therapeutic potential of targeting miR-34a has not yet been explored in the diabetic heart. Circulating levels of miR-34a were elevated in diabetic patients compared with controls [26], and was also shown to be upregulated in the hearts of type 2 diabetic patients with ischemic heart disease compared to the hearts of non-diabetic patients with ischemic heart disease [27]. Thus, miR-34a represents an unexamined target in a diabetic setting. The key aim of this study was to determine whether silencing miR-34a can improve diastolic function and attenuate cardiac fibrosis in the type 1 diabetic heart.

## 2. Materials and Methods

### 2.1. Animals, Induction of Diabetes and Experimental Outline

All experiments using animals were conducted in accordance with the Australian code for the care and use of animals for scientific purposes (National Health & Medical Research Council of Australia, 8th Edition, 2013). All animal procedures and care were approved by the Alfred Research Alliance Animal Ethics Committee (approval number E/1430/2014/B). Male mice on a FVB/N background were housed and bred onsite in a temperature-controlled environment under a 12 h light/dark cycle, and up to six littermates per cage. Mice had ad libitum access to standard rodent chow and water. Aged-matched (~six-week-old) littermate male mice were randomly allocated into diabetic and non-diabetic groups. Mice received five consecutive daily intraperitoneal (i.p.) injections of streptozotocin (STZ; 55 mg/kg in 0.02 mol/L citrate buffer, pH 4.5) to induce type 1 diabetes, or five consecutive daily intraperitoneal injections of citrate buffer vehicle of equivalent volume (non-diabetic group), and housed up to two littermates per cage. Mice were followed for 16 weeks, as previously described [28,29,30]. Diabetes was confirmed by measurements of blood glucose (via tail vein prick) every two to four weeks using a glucometer (blood glucose levels exceeding 28 mmol/L were considered diabetic). After eight weeks of untreated diabetes, mice were administered subcutaneously three consecutive daily doses of locked nucleic acid (LNA)-antimiR-34a (25 mg/kg per dose) or saline (150 μL). We have previously demonstrated that this dosing regime is sufficient to achieve knockdown of miRNAs in the heart for at least eight weeks [18,21,31,32]. Based on significant prior work, showing an LNA-control was not different to saline [31,33], control diabetic mice were administered saline rather than an LNA-control in the current study. In brief, the sequence of the LNA-control used in our prior miRNA studies [18,21,22] (5′-TcAtaCTatAtGaCA—3′ and 5′-TCATACTA—3′ (LNA uppercase, DNA lowercase) was checked against numerous databases and had no perfect match binding sites in the transcriptome [33]. The LNA-control was also validated in multiple assays (in vitro and in vivo) and shown not to differ from saline or untransfected/mock samples [31,33]. Three mice were administered LNA-antimiR-34a and 3 mice were administered saline at 10 weeks post diabetes induction due to an unexpected delay in the delivery of the LNA-antimiR-34a. However, this had no effect on knockdown of miR-34a gene expression (Supplementary Data Figure S1) and these mice were included in the study. After LNA oligonucleotide administration, mice were followed for a further eight weeks. A flowchart for the reporting of animal use, allocation, experimental analysis, follow up and exclusions following the CONsolidated Standards of Animal Experiment ReporTing (CONSAERT) guidelines as proposed by Weeks et al. [34] is provided in Supplementary Materials Figure S2.

### 2.2. Assessment of Hyperglycemia

Evaluation of hyperglycemia (>28 mmol/L) was performed every two to four weeks and at endpoint from blood collected from non-diabetic and diabetic mice. A tail vein prick was performed and glucose was measured using a glucometer (Accu-Chek^®^ Performa, Roche, Basel, Switzerland) [28,29,30]. The upper detection limit of the glucometer is 33.3 mmol/L. Therefore, readings at or above this value (recorded as HI on the glucometer) were given the upper value of 33.3 mmol/L. At endpoint, blood was collected by cardiac puncture in a heparinized syringe and glycated hemoglobin (HbA1c) was assessed using a Cobas b 101 machine (Roche, Basel, Switzerland). The normal range for HbA1c levels is between 4% and 5.6%, and under 42 mmol/mol. Mice were considered diabetic if mouse HbA1c levels were >6.5% and >48 mmol/mol. The lower detection limit of the Cobas b 101 machine is 4% and 20 mmol/mol.

### 2.3. LNA Oligonucleotides

The miRCURY LNA™ microRNA Inhibitor (mmu-miR-34a) is LNA™-enhanced (LNA/DNA mixmer with ~50% LNA content) and contains a phosphorothioate backbone (Qiagen, Hilden, Germany). The LNA-oligonucleotide was purified and analyzed using anion-exchange high-performance liquid chromatography, desalted and lyophilized as a sodium salt, and the identity of the compound was confirmed by mass spectrometry. The LNA-oligonucleotide was resuspended in saline at 5 mg/mL, aliquoted and stored at −20 °C. The sequence for mmu-miR-34a antimiRNA is 5′A*G*C*T*A*A*G*A*C*A*C*T*G*C*C 3′ (Batch Number 630532 and Lot number 235893690), where the phosphorothioate bonds are indicated by an asterix (*).

### 2.4. Left Ventricular (LV) Function

To obtain measures of LV diastolic function, echocardiography was performed in anesthetized mice (ketamine/xylazine/atropine, KXA: 80/8/0.96 mg/kg, i.p.) at baseline, eight weeks following STZ administration (pre-LNA antimiRNA administration) and after 16 weeks of diabetes (eight weeks post LNA antimiRNA administration and before tissue harvest), using a Vevo 2100 High Frequency Ultrasound System (Visual Sonics, Toronto, ON, Canada). Once mice were anesthetized, fur was removed using a depilatory cream, wiped clean and mice then placed on a Visualsonics handling platform in a supine position. Acoustic coupling gel was placed on the chest area and images acquired using a MS550D transducer. Core temperature was monitored using a rectal probe and maintained at the physiological level (36–37 °C). Evaluation of diastolic function was performed by echocardiography using measurement of transmitral flow parameters including the early (E) and late (A) diastolic filling velocities, the E/A ratio, from an apical four chamber view with pulsed wave Doppler. Tissue Doppler imaging was also performed to obtain early (e’) and late (a’) diastolic mitral annular velocity (e’/a’ ratio) and E/e’ ratio. At completion of echocardiography, mice were administered atipamezole (0.2 mg/kg, subcutaneously) to aid with recovery post KXA anesthesia. All echocardiography imagery was acquired by a single operator and analyzed blinded offline using the Vevo Lab Software (Version 3.2.6, Visual Sonics). All data were independently validated [35].

### 2.5. Tissue Collection

At study endpoint, mice were euthanized by exsanguination in anesthetized mice (KXA: 100:10:1.2 mg/kg, i.p.). Tissues (heart, lung, kidney, liver, spleen) were excised whole, weighed and snap frozen in liquid nitrogen and stored at −80 °C. The atria were removed from the hearts and weighed. Hind legs were removed, submerged in 1M NaOH and incubated at 37 °C for 7–10 h to remove surrounding tissue. Tibias were cleaned and rinsed in H_2_O then air dried. Determination of tibia bone length was measured using a Vernier caliper.

### 2.6. RNA Isolation

Total RNA was isolated from frozen mouse tissues using TRI Reagent (Sigma-Aldrich, St Louis, MO, USA). Briefly, mouse tissue was homogenized in 500 μL of TRI Reagent and centrifuged to remove high molecular weight components. The supernatant containing RNA was collected and chloroform added, and samples vortexed. Following centrifugation with chloroform, the upper aqueous phase was recovered and RNA collected by isopropanol precipitation and rehydration. RNA was quantified using the Implen**^®^** NanoPhotometer NP80 (Implen, Munich, Germany).

#### 2.6.1. Reverse Transcription

For miRNA gene expression analysis, 50 ng of total RNA was reverse transcribed using the TaqMan**^®^** MicroRNA Reverse Transcription Kit (Life Technologies, Carlsbad, CA, USA) according to manufacturer’s recommendations. The reverse transcription protocol consisted of 30 min at 16 °C, 30 min at 42 °C, and 5 min at 85 °C.

For gene expression analysis, 2 μg of total RNA was reverse transcribed using the High-Capacity cDNA Reverse Transcription Kit (Life Technologies, Carlsbad, CA, USA) according to manufacturer’s recommendations. The reverse transcription protocol consisted of 10 min at 25 °C, 120 min at 37 °C, and 5 min at 85 °C. Following cDNA synthesis, heart cDNA was diluted to a final concentration of 25 ng/uL.

#### 2.6.2. Quantitative RT-PCR (RT-qPCR)

MiRNA expression was assessed via RT-qPCR using an Applied Biosystems Quant Studio 7 Flex real-time PCR instrument. MiRNA expression was measured using the TaqMan**^®^** Universal Master Mix II, no UNG kit and TaqMan**^®^** MicroRNA Assays (20× primers; Life Technologies, Carlsbad, CA, USA; see Supplementary Materials, Table S1 for details) according to manufacturer’s recommendations. The PCR conditions consisted of 10 min at 95 °C; then 40 cycles of 15 s at 95 °C, and 60 s at 60 °C. Gene expression was normalized against snoU6 and data analyzed using the 2^−ΔΔCt^ algorithm.

For gene expression analysis, RT-qPCR was performed on an Applied Biosystems Quant Studio 7 Flex real-time PCR instrument. mRNA expression was measured using either the TaqMan**^®^** Fast Advanced Master Mix and TaqMan**^®^** Gene Expression Assays (Applied Biosystems, Foster City, CA, USA; 20X primer/probe mix, see Supplementary Materials, Table S2 for a list of assays) or SYBR™ Green PCR Master Mix and custom designed primers (Table S3) made by Integrated DNA Technologies (Coralville, IA, USA). The PCR conditions for TaqMan**^®^** fast assays consisted of 20 s at 95 °C, then 40 cycles of 1 s at 95 °C, and 20 s at 60 °C. The PCR conditions for SYBR™ Green Assays consisted of 2 min at 50 °C, 10 min at 95 °C, then 40 cycles of 15 s at 95 °C, and 1 min at 60 °C, and a melt curve stage of 15 s at 95 °C, 1 min at 60 °C and 15 s at 95 °C. All primers had a single peak in the melt curve and were 90–110% efficient on standard curves. Hypoxanthine phosphoribosyltransferase 1 (Hprt1) was used to standardize for cDNA concentration and all data were analyzed using the 2^−ΔΔCt^ method of quantification.

### 2.7. miR-34a Expression in Pure Single Cell Fractions from the Adult Mouse Heart

Data was mined from a prior study in which transcriptomic data was collected from adult mouse hearts subjected to a control/sham surgery or trans-aortic constriction. Pure single cell fractions of cardiac myocytes, cardiac fibroblasts and endothelial cells were obtained from retrograde collagenase-II perfusion, followed by pre-plating and sorting techniques [36]. Here, we mined data from adult male mice following 2 weeks of sham surgery as a representation of miR-34a expression in different cell types within the adult mouse heart under basal conditions (publicly available dataset GSE66974).

### 2.8. Histological Analysis

Heart samples were fixed in 4% paraformaldehyde overnight and paraffin embedded for histological analysis (Gribbles Veterinary Pathology, Clayton, Australia). Cardiac collagen deposition/interstitial and perivascular fibrosis was assessed by Masson’s trichrome stain on 6 μm cross-sections (Gribbles Veterinary Pathology, Clayton, Australia). Images were acquired using a light microscope (BX43, Olympus, Center Valley, PA, USA) at 40× magnification. Fibrosis was quantified using Image-Pro Plus 7 by assessing the number of blue pixels (collagen/fibrosis). The percentage of total fibrosis was calculated by dividing the blue-stained collagen tissue by the total area of the LV (combined fibrotic area and pink/red stained healthy cardiac tissue). The percentage of perivascular fibrosis was calculated by dividing the blue-stained collagen tissue around vessels by the total area of the LV. Interstitial fibrosis was calculated by subtracting the percent of perivascular fibrosis from the percent of total fibrosis. For assessment of myocardial capillary density (angiogenesis), 4 μm cross-sections were deparaffinized in xylene and rehydrated in a graded ethanol series, rinsed in dH_2_O and then washed in 1X PBS. Slides were blocked in a FITC Protein Blocking Agent (Thermo Fisher, Scoresby, Australia) for 1 h at room temperature, then co-stained with Alexa Fluor 568-conjugated isolectin B4 (Invitrogen I21412, 1:10 dilution, overnight at 4 °C) and FITC-conjugated wheat germ agglutinin (WGA; Vector Labs FL1021, 1:50 dilution, 2 h at room temperature). Sections were mounted with ProLong™ Gold antifade reagent (Life Technologies, Carlsbad, CA, USA) and visualized under an Olympus BX61 fluorescence microscope at 20× magnification. For each heart, capillary density was calculated by dividing the number of capillaries by the number of cardiomyocytes. Image acquisition and analysis were performed blinded to disease and treatment group.

### 2.9. Statistical Analyses

Statistical analyses were performed using GraphPad Prism (Version 8.1.2, San Diego, CA, USA). Results are presented as means ± SEM. Differences between groups were identified using one-way analysis of variance (ANOVA) followed by Fisher’s post hoc test or an unpaired *t*-test for comparing 2 groups alone, i.e., the non-diabetic and diabetic group. For blood glucose/HbA1c measurements, data were analyzed using a mixed-effects analysis with Fisher’s post hoc test, or a Kruskal–Wallis non-parametric one-way ANOVA with Dunn’s post hoc Test. For echocardiography parameters, differences between groups were identified using a two-way repeated measures ANOVA followed by Fisher’s post hoc test. A value of *p* < 0.05 was considered significant. All relative units are expressed as a fold change with the relevant control group normalized to 1.

## 3. Results

### 3.1. miR-34a Is Elevated in the Type 1 Diabetic Heart

**Figure 1:**
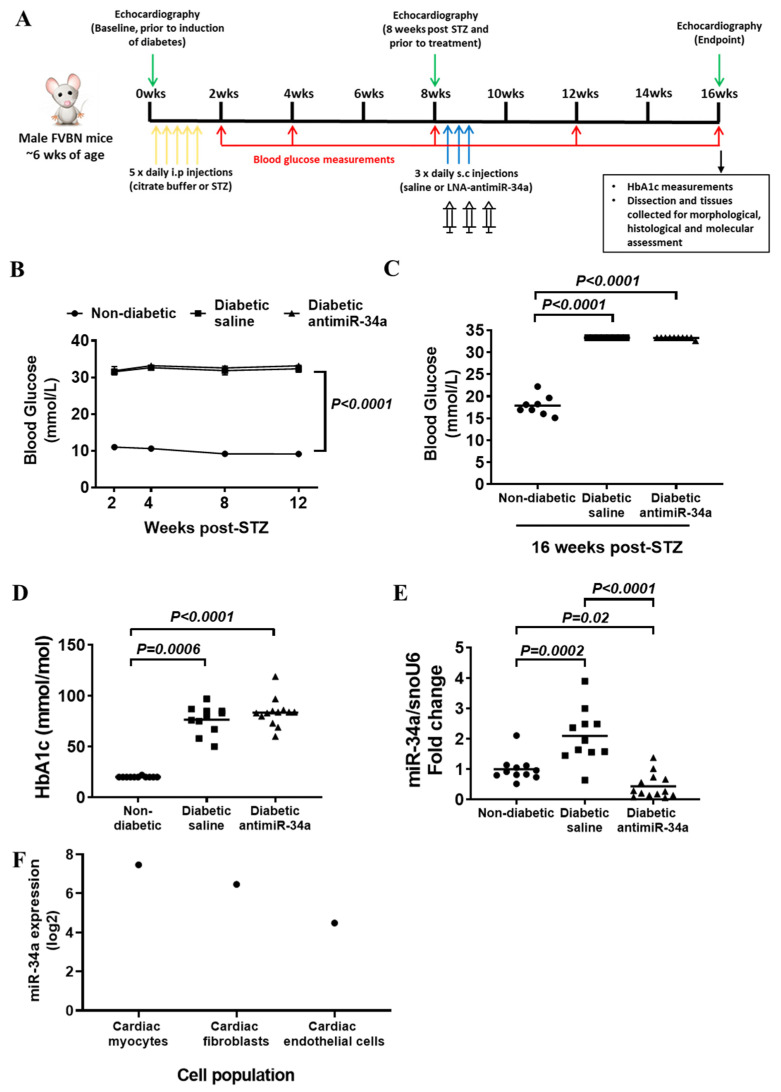
(**A**) Schematic showing the experimental timeline. Weeks (wks) on the timeline represent number of weeks post diabetes induction. (**B**) Blood glucose—time course (measured in conscious mice). Blood glucose levels were elevated in both diabetic saline and LNA antimiR-34a treated mice compared to non-diabetic mice after 2, 4, 8 and 12 weeks of diabetes. Data are presented as mean ± SEM and analyzed using a mixed-effects analysis with Fisher’s post hoc test. At 2 weeks N = 11 (non-diabetic, diabetic saline), 13 (diabetic LNA antimiR-34a), at 4, 8 and 12 weeks N = 8 (Non-diabetic, diabetic saline), 10 (diabetic LNA antimiR-34a). (**C**) Blood glucose at endpoint (measured in anesthetized mice). Blood glucose levels were significantly elevated at endpoint (i.e., after 16 weeks of diabetes) in both diabetic saline and LNA antimiR-34a treated groups compared to non-diabetic controls. Data analyzed using a Kruskal–Wallis non-parametric one-way ANOVA with Dunn’s post hoc Test. N = 8 (Non-diabetic, diabetic saline), 10 (diabetic LNA antimiR-34a). Lines indicate the mean. (**D**) Glycated hemoglobin levels (presented as IFCC standardization of HbA1c) were significantly elevated at endpoint (i.e., after 16 weeks of diabetes) in both diabetic saline and LNA antimiR-34a treated groups compared to non-diabetic controls. Data analyzed using a Kruskal–Wallis non-parametric one-way ANOVA with Dunn’s post- hoc Test. N = 11 (Non-diabetic, diabetic saline), 13 (diabetic LNA antimiR-34a). Lines indicate the mean. (**E**) Gene expression of miR-34a is elevated in diabetic hearts and attenuated with the LNA inhibitor. Quantification of miR-34a relative to snoU6 by qPCR in non-diabetic, diabetic saline and diabetic LNA antimiR-34a treated mice. Data analyzed using a one-way ANOVA with Fisher’s post hoc Test. N = 11 (non-diabetic, diabetic saline), 13 (diabetic LNA antimiR-34a). Lines indicate the mean. (**F**) miR-34a expression in isolated cardiac myocytes, endothelial and fibroblast cells in adult male mice following 2 weeks of a sham surgery. Data mined from the publicly available dataset GSE66974.

Following STZ injection, but prior to administration of LNA-antimiR-34a or saline (8 weeks post-STZ. Figure 1A), mice within the diabetic groups had elevated blood glucose compared to non-diabetic mice (Figure 1B). Compared to diabetic mice administered saline, administration of LNA-antimiR-34a had no impact on blood glucose during the study (Figure 1B) or at endpoint (Figure 1C). HbA1c levels were also comparably elevated in the diabetic groups compared to non-diabetic mice (Figure 1D and Table S4). miR-34a expression was elevated in the hearts of diabetic mice administered saline compared with non-diabetic mice. In contrast, miR-34a was significantly lower in hearts of diabetic mice administered LNA-antimiR-34a compared to both diabetic mice administered saline and non-diabetic mice (Figure 1E). Specific cardiac cell types were not isolated from diabetic hearts in the current study, but on mining a publicly available dataset (GSE66974) we present data from the adult male mouse heart to show that miR-34a is expressed in cardiac myocytes, cardiac fibroblasts and endothelial cells (Figure 1F).

### 3.2. Inhibition of miR-34a Had No Impact on Diabetes-Induced Diastolic Dysfunction

**Figure 2:**
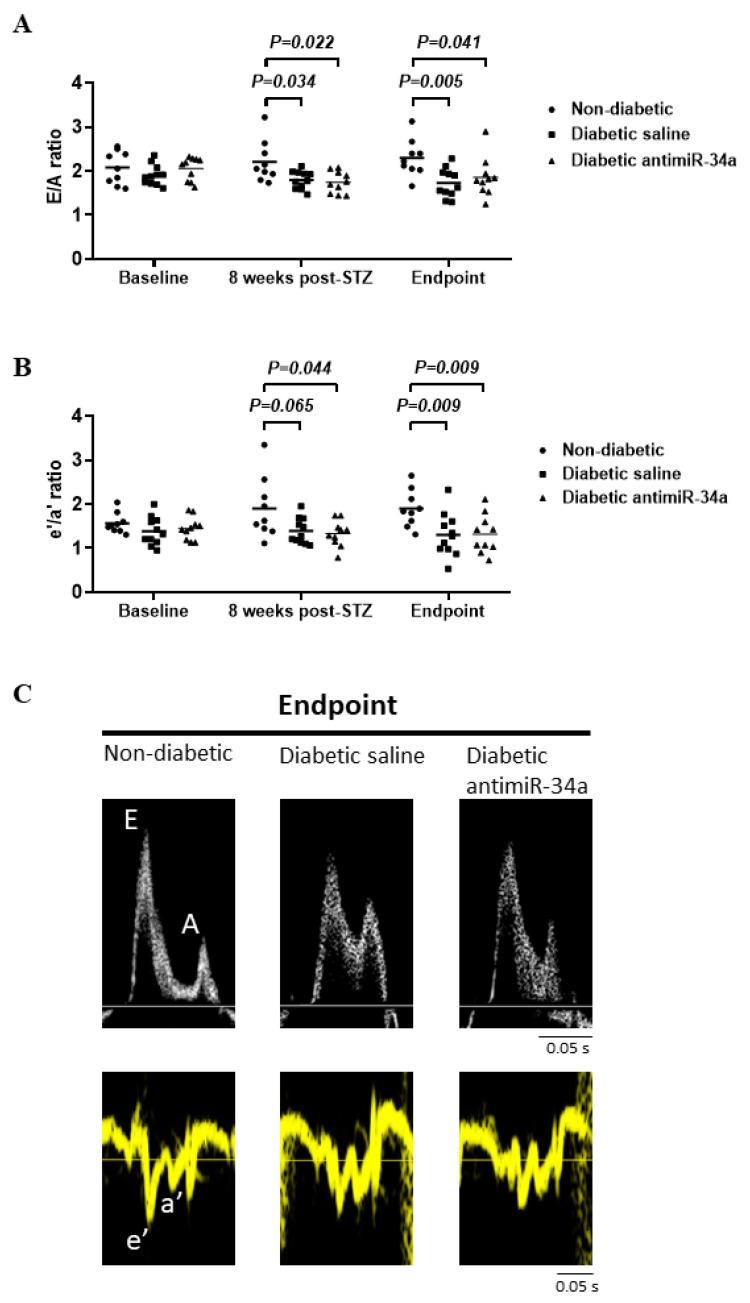
LNA antimiR-34a treatment was unable to improve established diabetes-induced diastolic dysfunction. Diabetic saline and LNA-antimiR-34a treated mice display diastolic dysfunction based on (**A**) E/A and (**B**) e’/a’ ratios following 8 and 16 weeks of diabetes (Endpoint). Data analyzed by two-way repeated measures ANOVA with Fisher’s post hoc test. Lines indicate the mean. N = 9 (non-diabetic), 11 (diabetic saline), 10 (diabetic LNA antimiR-34a). Mouse exclusions are outlined in the flowchart presented in Supplementary Materials, Figure S2. (**C**) Representative images of E, A, e’ and a’ waves from each group at endpoint.

Diastolic dysfunction is a characteristic feature of the diabetic heart. Diastolic function was assessed in diabetic mice 8 weeks post-STZ, and prior to administration of LNA-antimiR-34a or saline (Figure 1A). Diabetic mice displayed diastolic dysfunction prior to treatment based on lower E/A and e’/a’ ratios, a recognized feature of the STZ diabetic model [37,38,39] (Figure 2A,B). At study endpoint, both ratios remained lower in diabetic mice versus non-diabetic mice, and treatment with LNA-antimiR-34a had no significant effect (Figure 2A–C, Table S5).

### 3.3. Silencing miR-34a Had No Impact on Diabetes-Induced Changes in Organ Weights but Had a Mild Effect on Cardiac Enlargement and Ventricular BNP Gene Expression

**Figure 3:**
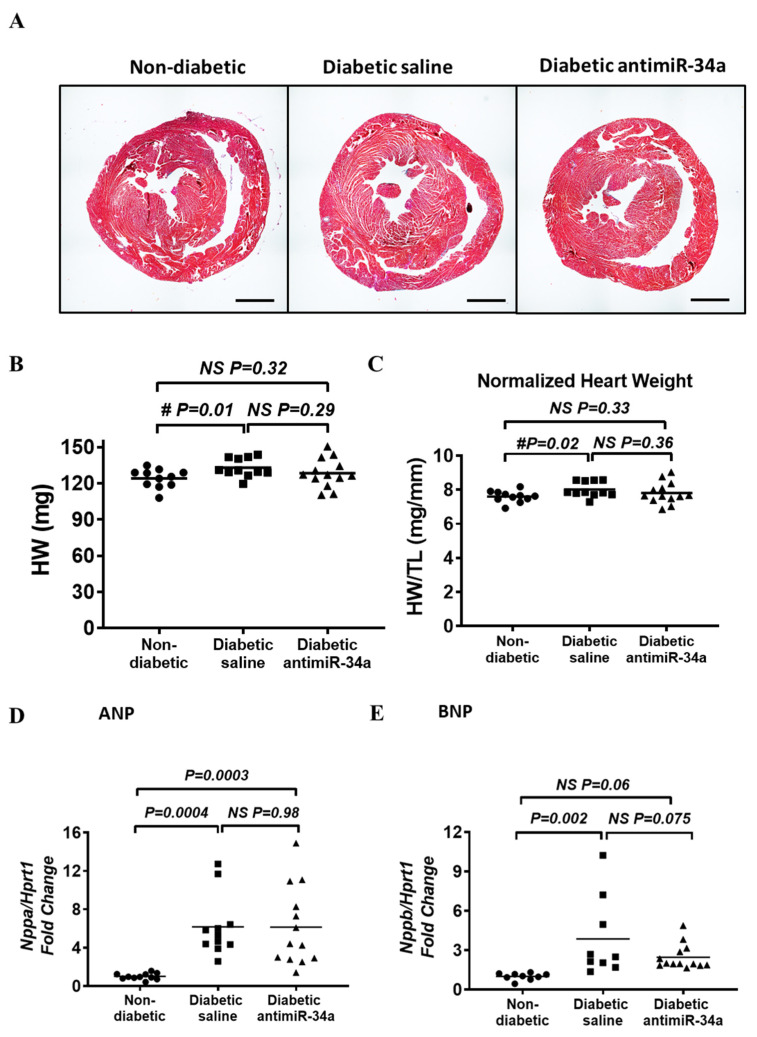
Inhibition of miR-34a had a modest effect on diabetes induced heart enlargement and BNP ventricular gene expression. (**A**) Representative ventricular cross-sections of non-diabetic and diabetic mice. Scale bar = 1 mm. (**B**,**C**) Heart weight (HW) and heart weight normalized to tibia length (TL). N = 11 (non-diabetic, diabetic saline), 13 (diabetic LNA antimiR-34a). No significant difference by ANOVA for panel (**B**,**C**). #*p*-value using unpaired *t*-test. Lines indicate the mean. (**D**,**E**) qPCR quantification of cardiac hypertrophy stress markers ANP (Nppa) and BNP (Nppb) gene expression relative to Hprt1. Data analyzed using a one-way ANOVA with Fisher’s post hoc Test. N = 11/9 for ANP/BNP for Non-diabetic, N = 11/9 for ANP/BNP for diabetic saline, N = 13 for ANP and BNP for diabetic LNA antimiR-34a (any exclusions are outlined in Supplementary Data Figure S2). Lines indicate the mean.

Diabetes was associated with an increase in normalized kidney weight, liver weight and spleen weight; consistent with a diabetic phenotype (Supplementary Materials, Table S4). These organ weights were not impacted by LNA-antimiR-34a treatment. There was no significant increase in heart weight in the diabetic model using one-way ANOVA (Table S4), but there was a small but significant increase in heart weight (unpaired *t*-test, *p* = 0.01, Figure 3A,B) and normalized heart weight when comparing the diabetic saline group to the non-diabetic group alone (unpaired *t*-test, *p* = 0.02, Figure 3C). Both of these modest increases in wet heart weight and HW/TL ratio were not apparent in the LNA-antimiR-34a treated diabetic mice compared to non-diabetic mice (unpaired *t*-test, *p* = 0.32 and *p* = 0.33, respectively, Figure 3B,C and Table S4). However, there were no significant differences between the two diabetic groups alone (unpaired *t*-test, *p* = 0.29 for heart weight and *p* = 0.36 for HW/TL, Figure 3B,C). Atrial weight was also elevated with diabetes, but inhibition of miR-34a had no impact (Table S4). Atrial natriuretic peptide (ANP) and B-type natriuretic peptide (BNP) ventricular gene expression were elevated with diabetes (Figure 3D,E), in accordance with our prior studies [37,38,39]. Inhibition of miR-34a had no impact on ANP expression, but appeared to have a modest effect on BNP ventricular gene expression. BNP was significantly elevated in the ventricles of diabetic saline mice versus non-diabetic mice (*p* = 0.002) but not in diabetic LNA-antimiR-34a treated mice versus non-diabetic mice (*p* = 0.06). There was no significant difference between the two diabetic groups (*p* = 0.075) (Figure 3E). There were no significant changes in gene expression of α-and β-MHC, and Serca2a (Supplementary Materials Figure S3A), or selected markers of oxidative stress, mitochondrial and inflammatory markers between diabetic mice administered saline or LNA-antimiR-34a (Supplementary Materials Figure S3B,C).

### 3.4. Inhibition of miR-34a Had a Modest Effect on Diabetes-Induced Fibrosis

**Figure 4:**
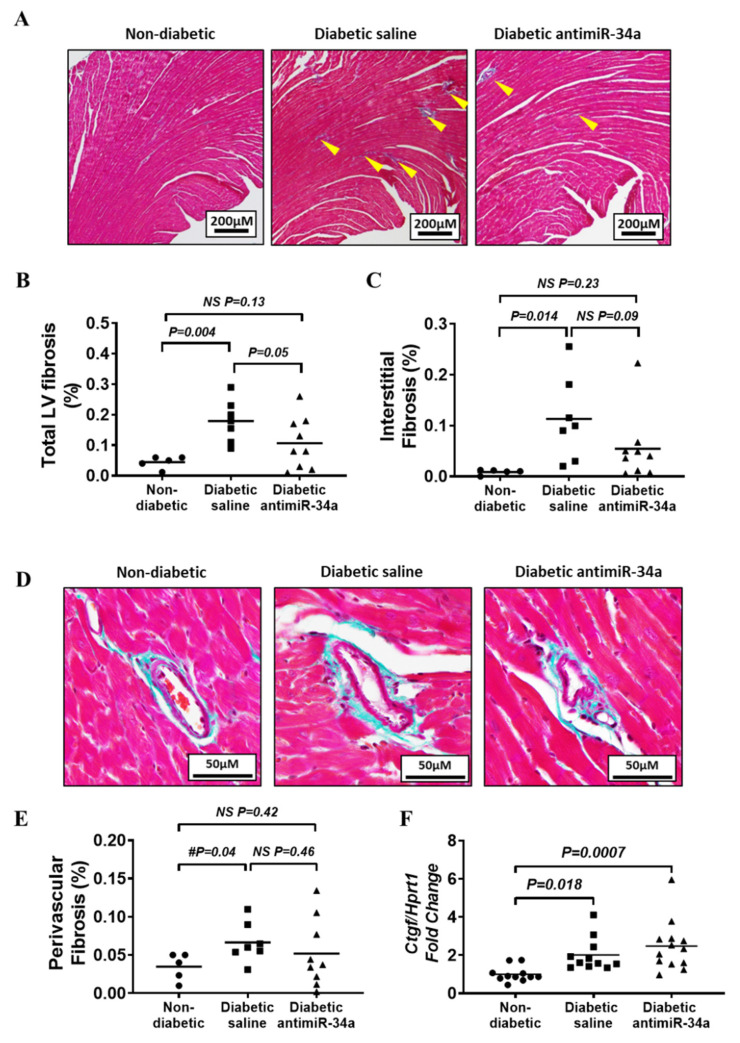
Inhibition of miR-34a limited diabetes-induced fibrosis. (**A**) Representative cross-sections of non-diabetic and diabetic saline and LNA antimiR-34a treated hearts stained with Masson’s trichrome. Scale bar = 200 µm. Yellow arrow heads highlight areas of fibrosis. (**B**) Quantification of total left ventricular (LV) fibrosis. (**C**) Quantification of interstitial LV fibrosis. For (**B**,**C**) Data analyzed using a one way ANOVA with Fisher’s post hoc test. N = 5 (non-diabetic), 7 (diabetic saline) and 9 (diabetic LNA antimiR-34a). Lines indicate the mean. (**D**) Representative cross-sections of non-diabetic and diabetic saline and LNA antimiR-34a treated hearts stained with Masson’s trichrome showing perivascular fibrosis. Scale bar = 50 µm. (**E**) Quantification of perivascular fibrosis. Data analyzed using an unpaired *t*-test. N = 5 (Non-diabetic), 7 (diabetic saline) and 9 (diabetic LNA antimiR-34a). Lines indicate the mean. (**F**) qPCR quantification of *Ctgf* gene expression relative to *Hprt1*. Data analyzed using a one way ANOVA with Fisher’s post hoc test. N = 11 (Non-diabetic, diabetic saline), 13 (diabetic LNA antimiR-34a). Lines indicate the mean. All data exclusions are outlined in the flowchart presented in Supplementary Figure S2.

Sixteen weeks of diabetes was associated with a modest increase in total LV fibrosis and interstitial fibrosis (Figure 4A–C). LNA-antimiR-34a prevented a significant increase in both total and interstitial fibrosis, and total LV fibrosis tended to be lower in the LNA-antimiR-34a treated diabetic mice (*p* = 0.05) (Figure 4A–C). Perivascular fibrosis was not significantly different between groups by one-way ANOVA, but there was a mild increase based on the result from an unpaired *t*-test comparing the diabetic saline and non-diabetic groups alone (Figure 4D,E). Connective tissue growth factor (CTGF) gene expression was elevated in diabetic mice but not reduced by LNA-antimiR-34a treatment (Figure 4F). There were no significant changes in gene expression of collagen 1, collagen 3, matrix metalloproteinase-2 (MMP-2), or tissue inhibitors of metalloproteinases (TIMPs 1–4) (Supplementary Materials Figure S4). This may not be surprising given fibrosis in this diabetic cardiomyopathy model is mild in comparison to other cardiac models such as pressure overload due to aortic constriction (Supplementary Materials Figure S5).

### 3.5. LNA-antimiR-34a Had No Significant Impact on the Diabetes-Induced Reduction in Capillary Density

**Figure 5:**
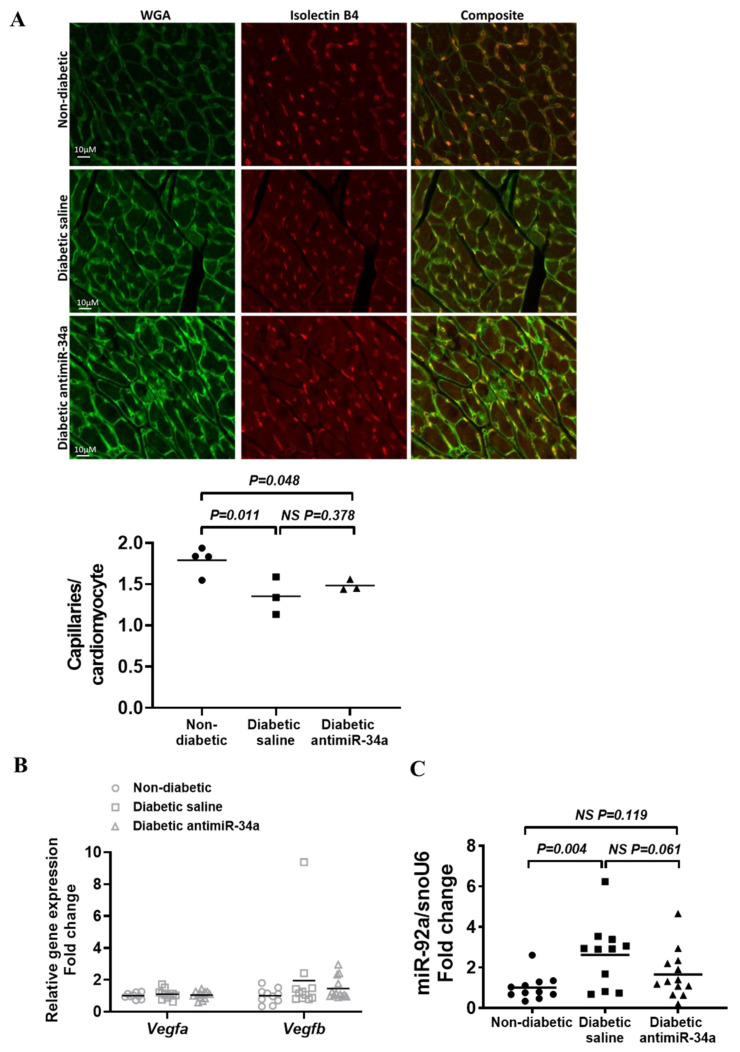
The effect of diabetes and LNA-antimiR-34a treatment on angiogenesis. (**A**) Representative cross-sections of the left ventricle, stained with wheat germ agglutinin (WGA) and isolectin B4, and quantification of capillary density. Scale bar = 10 μm. Data analyzed using one way ANOVA with Fisher’s post hoc test. N = 4 (Non-diabetic), 3 (diabetic saline, diabetic LNA antimiR-34a). Lines indicate the mean. (**B**) qPCR quantification of Vegfa and Vegfb relative to Hprt1. Data analyzed using one way ANOVA, no significant differences. N = 9 (Vegfa)/10 (Vegfb) for non-diabetic, 11 for diabetic saline, 13 for diabetic LNA antimiR-34a). Lines indicate the mean. (**C**) Quantification of miR-92a relative to snoU6 by qPCR in non-diabetic, diabetic saline and diabetic LNA antimiR-34a treated mice. Data analyzed using one way ANOVA with Fisher’s post hoc test. N = 11 (Non-diabetic, diabetic saline), 13 (diabetic LNA antimiR-34a). Lines indicate the mean.

A feature of the diabetic heart is reduced myocardial capillary density [5]. In the current study, capillary density was lower in hearts of diabetic mice administered saline compared to non-diabetic mice (*p* = 0.011, Figure 5A). Capillary density was also lower in LNA-antimiR-34a treated diabetic mice versus non-diabetic mice (*p* = 0.048), and not significantly different from diabetic saline mice (*p* = 0.378, Figure 5A). We also assessed the expression of genes known to play key roles in regulating vascular biology including vascular endothelial growth factor (VEGF [40]; target of miR-34a [41,42]) and miR-92a [42]. Vegfa and Vegfb gene expression were not significantly altered in diabetic hearts compared to non-diabetic hearts (Figure 5B). Expression of miR-92a was elevated in hearts from control diabetic mice (*p* = 0.004) but not LNA-antimiR-34a treated diabetic mice (*p* = 0.119), but there was no significant difference between the two diabetic groups (*p =* 0.061).

### 3.6. LNA-antimiR-34a Treatment Had Little Impact on miR-34a Target Genes

**Figure 6:**
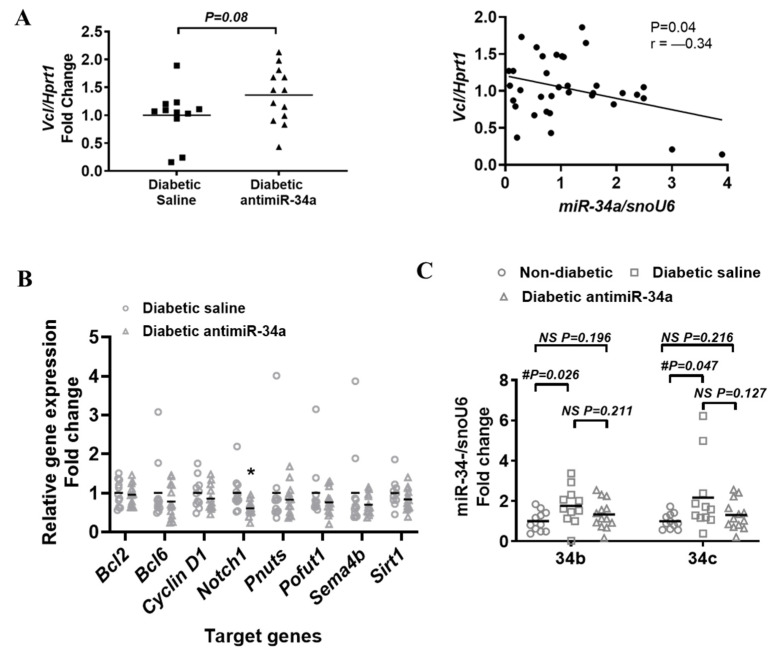
The effect of diabetes and LNA-antimiR-34a treatment on the expression of target mRNAs and miR-34b and -c. (**A**) Left: qPCR quantification of Vinculin (Vcl), a target gene of miR-34a relative to Hprt1 in diabetic saline and diabetic LNA-antimiR-34a treated hearts. Data analyzed using an unpaired *t*-test, N = 11 for diabetic saline, 13 (diabetic LNA antimiR-34a). Lines indicate the mean. Right: Expression of Vcl and miR-34a gene expression in diabetic saline and diabetic LNA-antimiR-34a treated hearts is inversely correlated. N = 25, Pearson correlation. (**B**) qPCR quantification of miR-34a target genes relative to Hprt1. Data analyzed using an unpaired *t*-test. N = 11 for diabetic saline (except for Sirt1, N = 10), 13 for diabetic LNA antimiR-34a (except for Notch1, N = 12). Lines indicate the mean. * *p* < 0.05. (**C**) Quantification of miR-34b and -c relative to snoU6 by qPCR in non-diabetic, diabetic saline and diabetic LNA antimiR-34a treated mice. Data analyzed using unpaired *t*-test (#). N = 11 (Non-diabetic, diabetic saline), 13 (diabetic LNA antimiR-34a). Lines indicate the mean.

To explore potential reasons as to why inhibiting miR-34a provided limited protection in the diabetic heart, we first examined the expression of miR-34 target genes, some of which were previously shown to be regulated in settings of cardiac protection (vinculin (Vcl), Bcl2, Bcl6, cyclin D1, Notch1, Pnuts, Pofut1, Sema4b, Sirt1) [22,23]. Vcl which plays a critical role for protecting the heart against cardiomyopathy [43] tended to be greater in hearts from LNA-antimiR-34a treated diabetic mice than control diabetic mice (*p* = 0.08) and there was a significant inverse relationship between miR-34a expression with Vcl expression (Figure 6A). However, there was no obvious regulation of any other miR-34a target genes in the diabetic heart, besides Notch1 which was unexpectedly decreased rather than elevated (Figure 6B).

Next, we assessed the expression of miR-34b and miR-34c. In models of severe cardiac pathology in which antimiR-34a provided little or no protection, we identified upregulation of miR-34b and miR-34c [18,21,22]. In the current study, there were no significant differences in miR-34b or miR-34c assessed by one way ANOVA. However, when comparing expression of miR-34b and miR-34c in the diabetic saline group versus the non-diabetic control group alone with an unpaired *t*-test, miR-34b and -34c were elevated (*p* = 0.026 and *p* = 0.047, respectively) (Figure 6C).

## 4. Discussion

**Figure 7:**
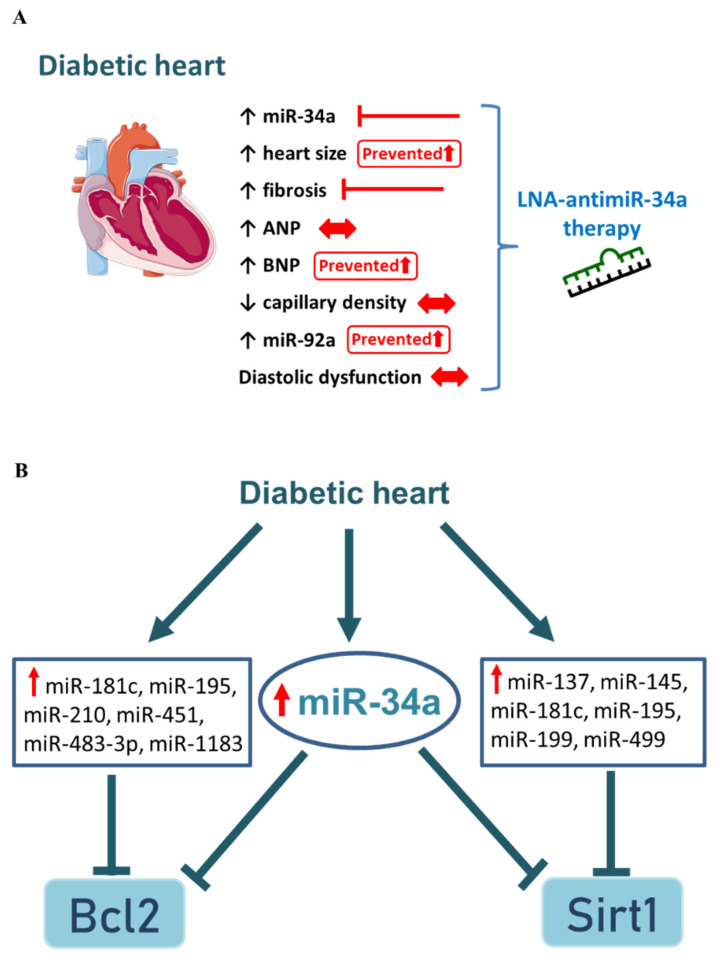
Diabetes-induced cardiomyopathy is a complex disorder. (**A**) Summary of the major findings in the current study in the heart in response to STZ-induced type 1 diabetes and the impact of LNA-antimiR-34a treatment. (**B**) Schematic demonstrating complex signaling of miRNAs and their target genes in diabetic cardiomyopathy.

The major goal of this study was to assess whether therapeutically targeting miR-34a in the diabetic heart with established diastolic dysfunction would improve diastolic function and prevent cardiac fibrosis. To our knowledge, this is the first study to inhibit miR-34a in the diabetic heart in vivo. The key findings to come from this study are summarized in Figure 7A. Despite, elevation of miR-34a in the diabetic heart, inhibition of miR-34a had no significant impact on diastolic function, and only modestly attenuated cardiac enlargement, fibrosis, and ventricular gene expression of BNP, and an anti-angiogenic miRNA (miR-92a). One miR-34a target, vinculin, was inversely correlated with miR-34a expression in the diabetic heart, but several other miR-34a targets were unchanged.

In previous work from us and other investigators, it had been shown that inhibiting miR-34a provided cardiac protection in settings of moderate cardiac pathology including aging [23], a model of moderate pressure overload-induced hypertrophy [18], acute myocardial infarction [23], and dilated cardiomyopathy [21]. However, we also showed that inhibiting miR-34a in more severe cardiac models of chronic myocardial infarction [22], severe pressure overload [18], and dilated cardiomyopathy with atrial fibrillation [21] provided little or no advantage. These severe cardiac models displayed significantly more fibrosis and systolic dysfunction than the models of moderate cardiac pathology. It was concluded that in more severe heart disease models, inhibiting miR-34a alone was inadequate to attenuate or reverse established severe pathology. The STZ model of diabetic cardiomyopathy used in the current study was considered to represent a mild-to-moderate model of cardiac pathology since it is associated with diastolic dysfunction, moderate cardiac fibrosis and modest elevation of ventricular BNP and/or ANP [37,38,39]. Thus, we hypothesized that silencing miR-34a in the diabetic heart would provide substantial benefit. A number of factors may explain why inhibition of miR-34a was unable to restore diastolic function in the diabetic heart, and only had a limited or no impact on other features including hypertrophy, fibrosis, capillary density, and cardiac gene expression. First, diabetes represents a systemic disorder impacting multiple organs/systems within the body. By contrast, in our prior studies in which inhibiting miR-34a was able to improve systolic function, we used models in which the heart was specifically targeted, i.e., surgical interventions targeting the heart alone or cardiac-specific transgenic mouse models. The systemic nature of diabetes is associated with the dysregulation of multiple miRNAs in numerous tissues and circulation, as well as inter-organ cross talk [44]. In the current study, kidney, spleen and liver weights were elevated with diabetes. Thus, targeting diabetes with miRNA-based drugs may be more challenging than targeting pathologies which are more limited to one tissue type. Second, targeting a single miRNA in the diabetic heart may be insufficient to improve heart function in a setting of established dysfunction. In our prior work, we found that miR-34b and miR-34c were elevated in addition to miR-34a in settings of substantial pathology due to severe pressure overload or chronic myocardial infarction [22,45], and that inhibiting the miR-34 family (i.e., miR-34a, -34b and -34c) attenuated pathology/dysfunction but inhibiting miR-34a alone did not [18,22]. Both miR-34b and miR-34c were elevated in hearts of diabetic mice versus non-diabetic mice, but not in hearts from LNA-antimiR-34a treated mice. Thus, any additional protection from targeting the miR-34 family in the diabetic heart is considered to be minimal. Rather, it may be necessary to target other miRNAs which play key functional roles in the diabetic heart such as miR-133a, miR-195, miR-30c, miR-200b and miR-15a/b [46,47,48]. A profiling study on LV samples from an STZ-induced diabetic mouse model showed that over 300 miRNAs were differentially regulated and over 200 were upregulated by greater than 2-fold [49]. This may explain, at least in part, why LNA-antimiR-34a treatment in the current study did not regulate several miR-34a targets genes known to contribute to cardiac protection including Bcl2, Pnuts, Sirt1, Vegfa and Vegfb. For example, of the upregulated miRNAs described in previous diabetic studies, a number including miR-195, miR-483-3p, miR-210, miR-451, miR-181c, miR-1183, miR-145, miR-199, miR-137 and miR-499 have been shown to regulate mRNAs that are recognized targets of miR-34a including Bcl2 and Sirt1 [27,50,51,52,53,54,55,56,57,58] (Figure 7B). It is therefore probably not surprising that in a setting of diabetes in which multiple miRNAs are upregulated and targeting the same mRNAs, inhibiting miR-34a alone had no significant impact on the regulation of some target genes (Figure 7B). This may also explain a prior report in the human diabetic heart, in which one miR-34a target was regulated but another two targets were not [27]. Third, we previously demonstrated that anitmiR-34 could regulate the expression of other miRNAs in addition to miR-34 by binding to miR-34 direct target genes which also play roles as transcription factors, and hence regulating other miRNAs with other targets [59]. A relevant example to the diabetic heart is that antimiR-34 differentially regulated miR-15a and miR-15b [59], which have been implicated in playing a role in the diabetic heart [48]. Finally, the timing of intervention may also be important. We chose to intervene at a time point with established cardiac dysfunction. Katare and colleagues showed that circulating and cardiac miR-34a were elevated in type 2 diabetic patients prior to any obvious systolic or diastolic dysfunction. This suggests that miR-34a is upregulated in the early stages of disease [27]. Thus, an earlier intervention may be more advantageous in attenuating diastolic dysfunction. However, in a clinical setting, an intervention would need to be commenced based on elevated circulating levels of miR-34a rather than the presence of any cardiac dysfunction. This may be challenging given that circulating levels of miR-34a can be altered by other disease states and conditions including chronic migraine [60] and non-alcoholic fatty liver disease [61].

While silencing miR-34a was unable to restore diastolic dysfunction in a setting of established dysfunction, there was evidence that inhibition of miR-34a had the potential to attenuate diabetes-induced total and interstitial cardiac fibrosis. Consistent with this result, a previous in vitro study demonstrated that a miR-34a inhibitor could suppress TGFβ-induced proliferation of rat cardiac fibroblasts [62]. We also showed that antimiR-34a attenuated fibrosis in a mouse model with dilated cardiomyopathy [21]. However, of note, other miRNAs have been implicated in regulating fibrosis in the diabetic heart including miR-133a [63], miR-200b [64], and miR-15a/b [48]. Inhibiting miR-34a also appeared to have a modest impact on preventing diabetes-induced cardiac enlargement and BNP expression, but no effect on ANP gene expression. This could be in part because two miRNAs shown to play a key role in repressing ANP mRNA in human embryonic stem cell-derived cardiomyocytes (miR-425 and miR-155; [65]), were significantly decreased in the STZ-induced diabetic mouse heart [49]. In the current study, myocardial capillary density was reduced in the diabetic heart, regardless of treatment. At the molecular level, miR-92a was significantly elevated in hearts of diabetic control mice but not diabetic antimiR-34a treated mice, but other regulators of vascular biology (Vegfa and Vegfb) were unchanged. miR-34a and miR-92a are recognized to have anti-angiogenic properties via the down-regulation of a shared target, Sirt1 [66,67,68]. MiR-34a was also shown to promote vascular smooth muscle cell calcification in the aorta of mice, and miR-34a knockout mice were protected [66]. The absence of any significant vascular protection in diabetic antimiR-34a treated mice may be related to the absence of any significant changes in Sirt1, Vegfa and Vegfb. Collectively our data and previous literature suggest a drug targeting multiple miRNAs may offer more protection.

In reviewing the literature, there is limited evidence of any miRNA-based therapeutic or intervention restoring or improving cardiac function of the diabetic heart, i.e., assessment of an intervention in a setting of established diastolic dysfunction. Examples of miRNAs considered to represent therapeutic targets have included miR-133a, miR-195, miR-30c, miR-200b and miR-15a/b [46,47,48]. However, within these studies, restoration of miRNAs were undertaken in in vitro settings rather than in vivo [48,69], or for the in vivo assessments, the miRNA intervention (therapeutic/transgenic) occurred simultaneously with the pathology (i.e., a prevention study), or it was unclear whether there was established cardiac dysfunction prior to treatment/miRNA intervention [50,64,70,71]. There has been intense interest in developing miRNA-based drugs for various diseases including the diabetic heart [46,47]. To be successful in a diabetic setting with established cardiac dysfunction and fibrosis, it is likely that targeting multiple miRNAs will be required. In developing strategies targeting multiple miRNAs, the role of miRNAs in other tissue and cell types will also need to be considered. For example, a therapy targeting miR-34 was used in a clinical trial for liver cancer [72]. However, in the context of cancer, the strategy is to increase miR-34 rather than silence it. Given miR-34 and other miRNAs are ubiquitously expressed, it will be important to target organs or cell types specifically, for example with antibody or vector-based approaches [12,73].

The application of LNA-based antimiR therapies in preclinical animal models has been established in multiple disease settings [74,75,76], and recently in a setting of diabetes-induced heart disease and cardiac dysfunction [77]. Several studies have demonstrated efficacy and safe use of LNA-based antimiR therapies in human clinical trials, including miravisen (targeting the liver expressed miR-122 for the treatment of hepatitis C [13]), cobomarsen (an inhibitor of miR-155 in patients with T-cell lymphoma [78]), and MRG-110 (an inhibitor of miR-92a-3p, which is known to have a role in cardiovascular disease and wound healing [79]). However, delivery of LNA-antimiRs to the heart still poses some challenges. Further strategies to improve specificity, uptake and targeted delivery of antimiR therapies will be required [76,80,81]. One emerging technology to deliver miRNA inhibitors to heart tissue is the use of ultrasound and a microbubble-targeted delivery system [82]. This has proven to be an effective technique to deliver LNA-antmiR-23a to the heart in a mouse model of pathological cardiac hypertrophy [82].

*Limitations and future work:* There are some limitations to our work which could form the basis of future work. Firstly, we showed that miR-34a was elevated in hearts of diabetic mice and reduced in hearts of LNA-antimiR-34a treated diabetic mice, and that miR-34a is expressed in mouse cardiac myocytes, cardiac fibroblasts and endothelial cells, but we did not assess whether LNA-antimiR-34a reduced miR-34a in each of these cell types. Second, we did not measure the level of circulating miRNA-34a in the plasma of non-diabetic and diabetic mice. Circulating miRNAs have been proposed as biomarkers in the prognosis of cardiac disease, as they reflect the progression and severity of the disease state. A previous study demonstrated a correlation between cardiac and circulating miR-34a levels in human diabetic hearts, and suggested the elevated circulating miR-34a may be of cardiac origin [27]. Thirdly, we did not investigate the effect of miR-34a inhibition on proliferation. In cancer cells, it is well recognized that miR-34a inhibits cellular proliferation [83]. In the hearts of neonatal mice, overexpression of miR-34a limited cardiomyocyte proliferation and regeneration following cardiac injury [20]. In a diabetic setting, in vitro studies suggest that inhibition of miR-34a may have differential effects depending on cell type. Inhibition of miR-34a decreased high glucose induced cell death in cultured human adult cardiomyocytes, whereas in human cardiac progenitor cells isolated from the diabetic heart, inhibition of miR-34a reduced proliferation via a mechanism involving the miR-34a targets, cyclin D1, Bcl2 and Sirt1 [27]. While, we did not directly assess the effect of miR-34a inhibition on proliferation in the diabetic heart, the expression of cyclin D1, Bcl2 and Sirt1 were unchanged by LNA-antimiR-34a treatment. Finally, when cardiac fibrosis is mild, as observed in our model of diabetic cardiomyopathy, it will be challenging to identify significant changes in any one gene with interventions. To explore the potential antifibrotic actions of antimiR-34a or other miRNAs it would be advisable to study a model with more fibrosis.

## 5. Conclusions

In summary, despite LNA-antimiR-34a preventing a diabetes-induced increase in miR-34a in the mouse heart, inhibition of miR-34a was unable to improve diastolic function in a setting of established diastolic dysfunction, and only had a modest effect on attenuating diabetes-induced cardiac enlargement, cardiac fibrosis and elevation of ventricular BNP. Since the expression of a number of miRNAs are elevated in the diabetic heart and share target mRNAs with miR-34a, strategies targeting multiple miRNAs and/or earlier intervention is likely to be required to achieve significant functional benefit.

## Supplementary Materials

The following supporting information can be downloaded at: https://www.mdpi.com/article/10.3390/cells11193117/s1, Figure S1: Gene expression of miR-34a in non-diabetic, diabetic saline and diabetic antimiR-34a groups at endpoint; Figure S2: Flowchart reporting animal exclusions and experimental analysis; Figure S3: Gene expression of cardiac contractile genes, oxidative stress, mitochondrial and inflammatory markers; Figure S4: Gene expression of collagens, MMP2 and tissue inhibitors of metalloproteinases genes in hearts of non-diabetic and diabetic mice; Figure S5: Comparison of fibrosis in a mouse model of diabetic cardiomyopathy and pressure overload due to transverse aortic constriction (TAC); Table S1: microRNA primer sequences; Table S2: mRNA primer details- Taqman; Table S3: mRNA primer details-Sybr; Table S4: Morphological and systemic characteristics of non-diabetic and diabetic mice; Table S5: Pulsed waved and tissue Doppler echocardiography data in non-diabetic and diabetic mice.
