# Supplementary material for: In Vivo Inhibition of miR-34a Modestly Limits Cardiac Enlargement and Fibrosis in a Mouse Model with Established Type 1 Diabetes-Induced Cardiomyopathy, but Does Not Improve Diastolic Function
Source: Cells. 2022 Oct 3;11(19):3117. doi: 10.3390/cells11193117 (PMC9563608; doi:10.3390/cells11193117)
Supplement: Supplementary file 1 [file cells-11-03117-s001.zip › cells-1909458-supplementary.pdf]

Figure S1: Gene expression of miR-34a in non-diabetic, diabetic saline and diabetic antimiR-34a groups at endpoint.

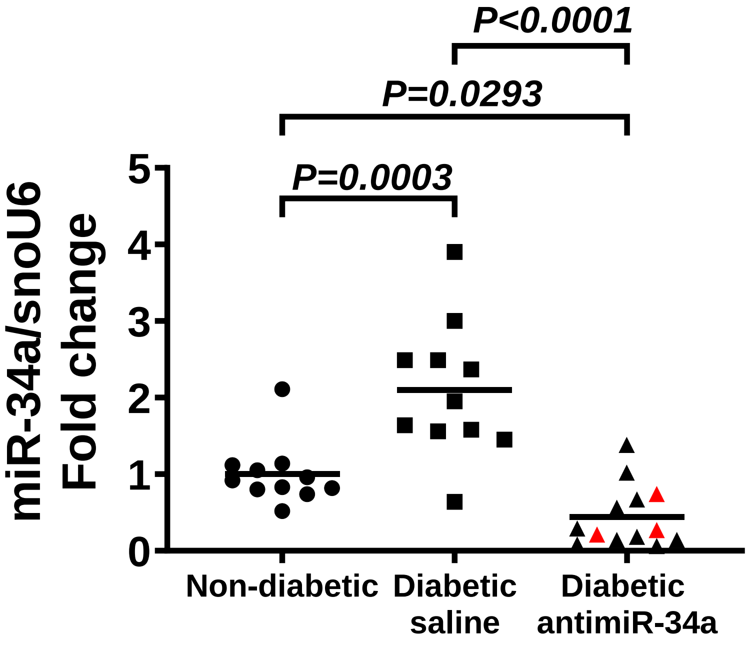

Figure S1: Quantification of miR-34a relative to snoU6 by qPCR in non-diabetic, diabetic saline and diabetic LNA antimiR-34a treated mice at endpoint. The 3 mice that received the LNA-antimiR-34a later (highlighted in red tringles) are indistinguishable from the other mice. Data analyzed using a one way ANOVA with Fisher’s post-hoc Test. N=11 (Non-diabetic, diabetic saline), 13 (diabetic LNA antimiR-34a). Lines indicate the mean.

Figure S2: Flowchart reporting animal exclusions and experimental analysis

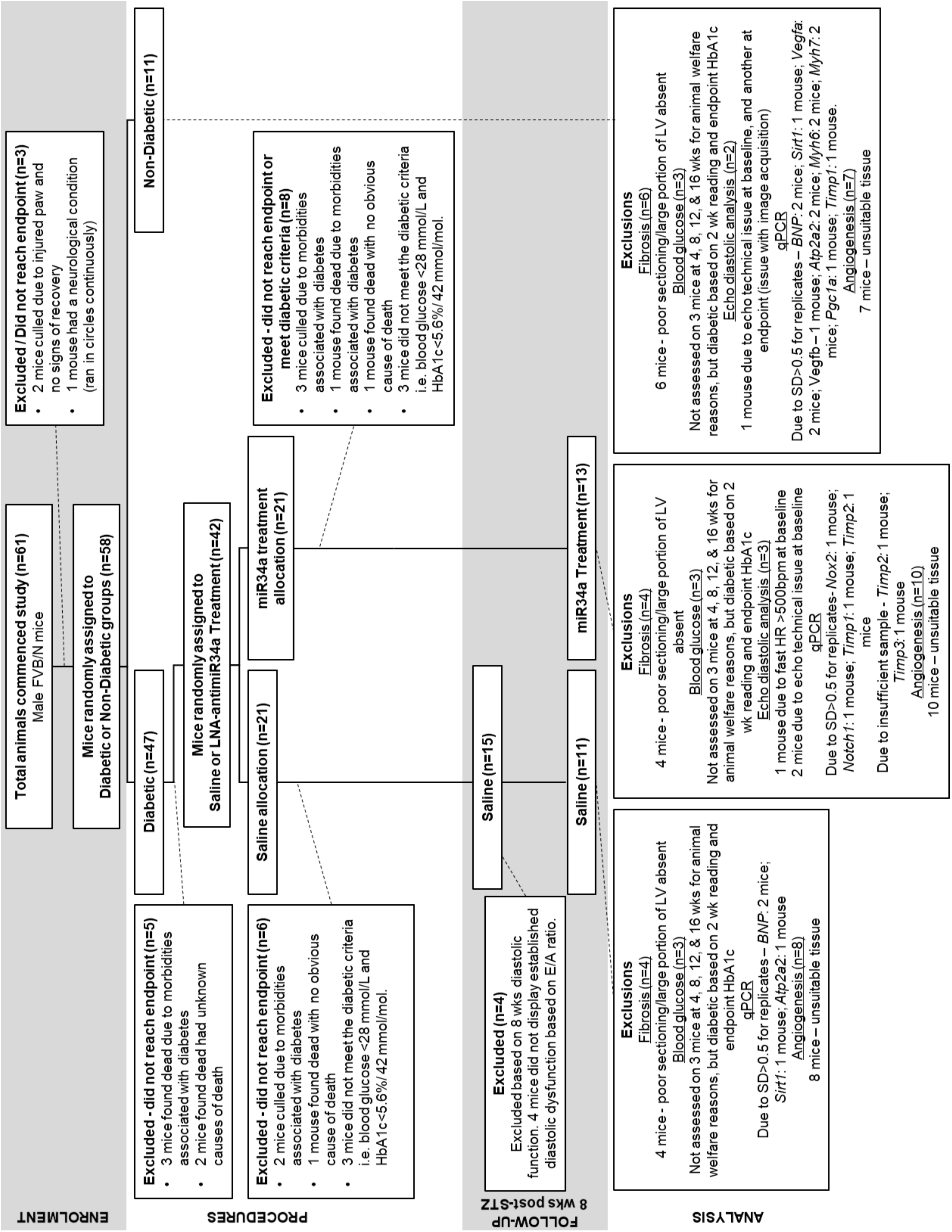

Figure S3: Gene expression of cardiac contractile genes, oxidative stress, mitochondrial and inflammatory markers

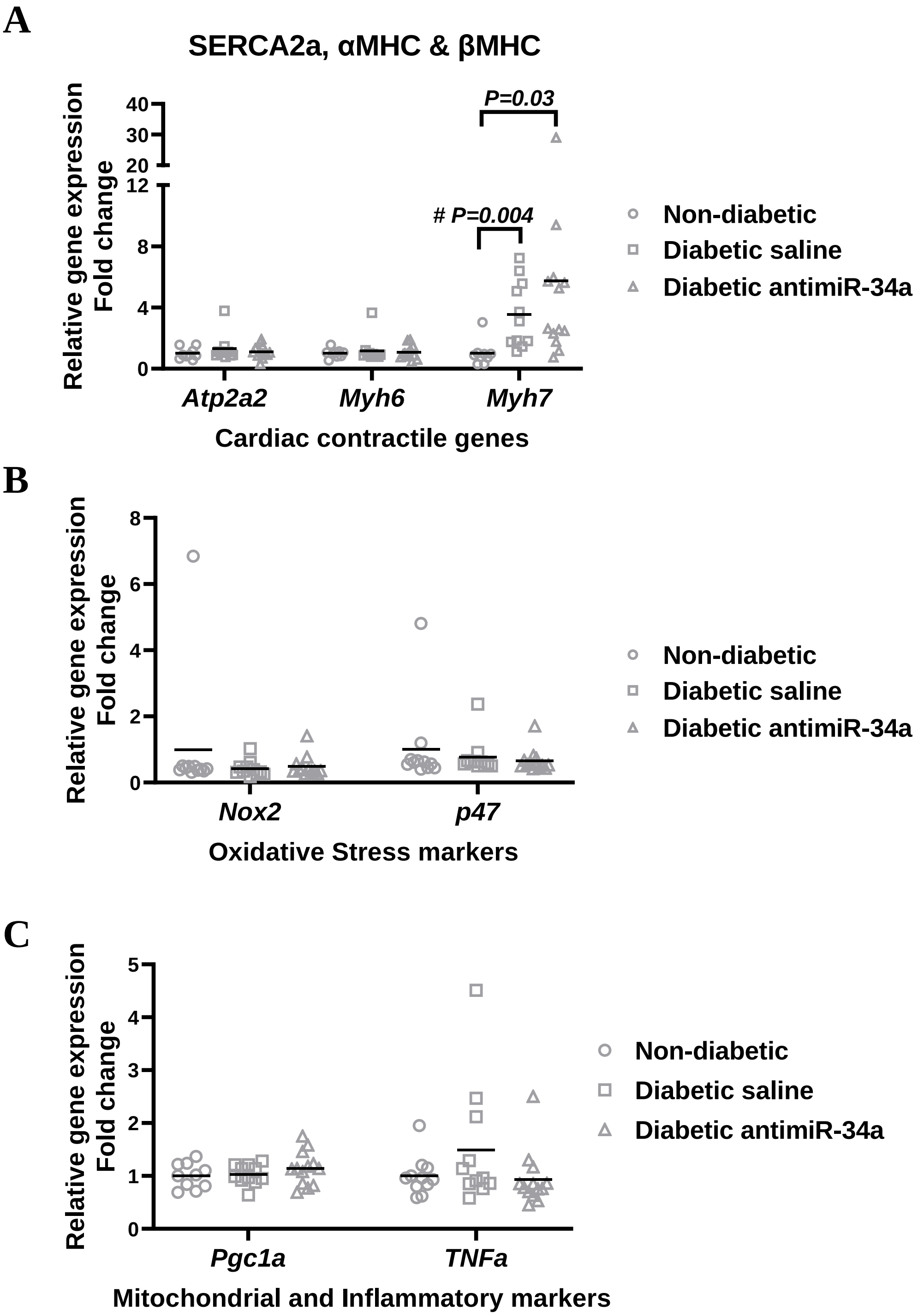

Figure S3: Gene expression of cardiac contractile genes, oxidative stress, mitochondrial and inflammatory markers in non-diabetic and diabetic mice after 16 weeks of diabetes and following LNA antimiR-34a treatment. A) qPCR quantification of cardiac contractile genes *Serca2a (Atp2a2),* α-myosin heavy chain *(Myh6)* and β-myosin heavy chain *(Myh7)* gene expression relative to *Hprt1*. Data analyzed using a one way ANOVA with Fisher's post-hoc Test. #P-value using unpaired t-test. N=9 for Non-diabetic, N=11 for diabetic saline (except for Serca2a, N=10), N=13 for diabetic LNA antimiR-34a. Lines indicate the mean. **B & C)** Gene expression of oxidative stress, mitochondrial and inflammatory markers. qPCR quantification of *Nox2, p47, Pgc1a*, and *TNFa* expression relative to *Hprt1*. Data analyzed using a one way ANOVA with Fisher's post-hoc Test. N=11 for non-diabetic (except for *Pgc1a*, N=10), N=11 for diabetic saline, N=13 for diabetic LNA antimiR-34a (except for *Nox2,* N=12). Exclusions are outlined in Fig S2. Lines indicate the mean.

Figure S4: Gene expression of collagen and tissue inhibitors of metalloproteinases genes in hearts of non-diabetic and diabetic mice

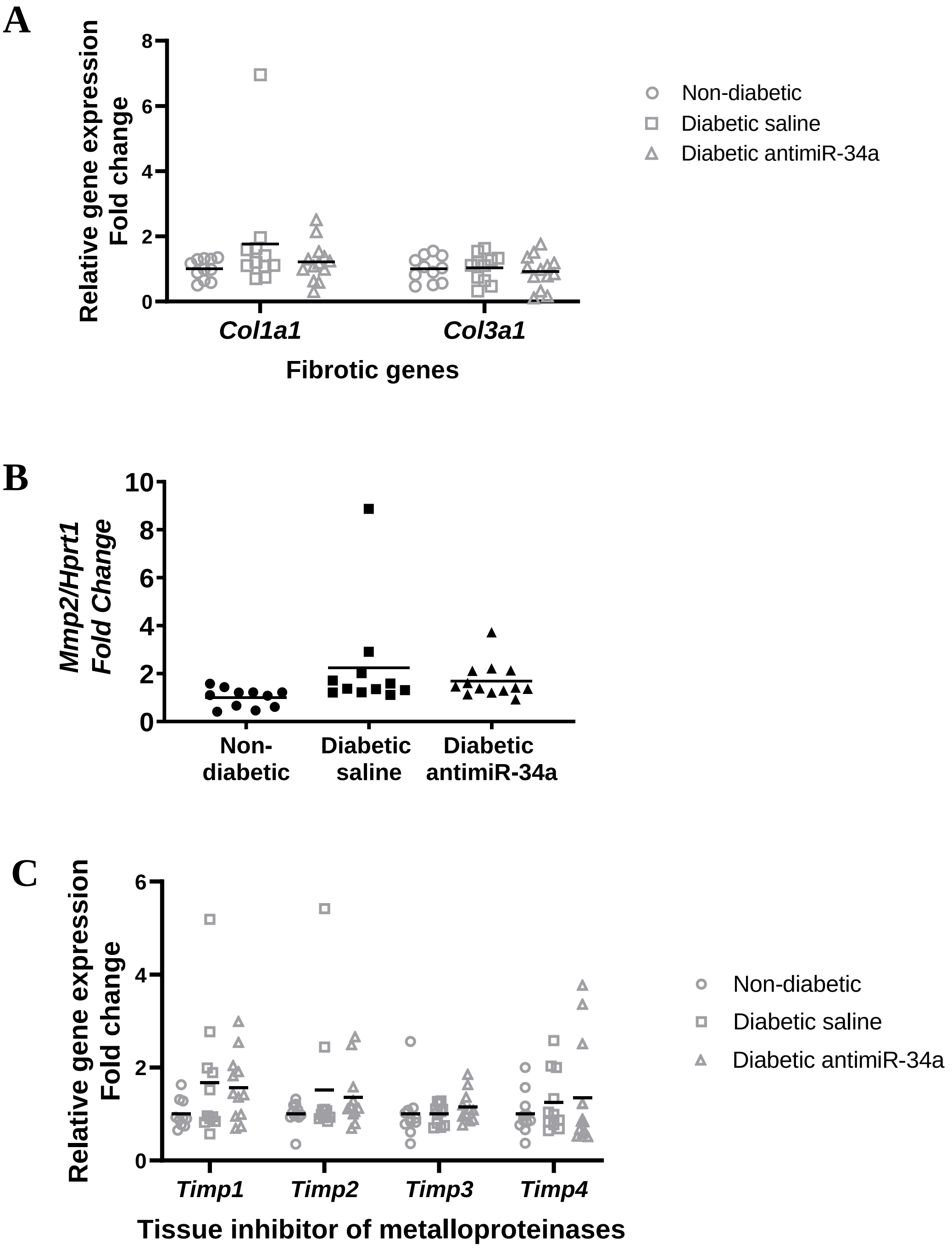

Figure S4: Gene expression in hearts of non-diabetic and diabetic mice after 16 weeks of diabetes and following LNA antimiR-34a treatment. A) qPCR quantification of *Col1a1* and *Col3a1* gene expression relative to *Hprt1*. **B)** qPCR quantification of *Mmp2* gene expression relative to *Hprt1*. **C)** qPCR quantification of *Timp1, Timp2, Timp3 and Timp4* gene expression relative to *Hprt1*. **A-C)** Data analyzed using a one way ANOVA with Fisher's post-hoc Test. Lines indicate the mean. N=11 for non-diabetic (except for *Timp1*, N=10), N=11 for diabetic saline, N=13 for diabetic LNA antimiR-34a (except for *Timp1*, N=12; *Timp2* and *Timp3*, N=11). Exclusions are outlined in Fig S2. Lines indicate the mean.

Figure S5: Comparison of fibrosis in a mouse model of diabetic cardiomyopathy and pressure overload due to transverse aortic constriction (TAC).

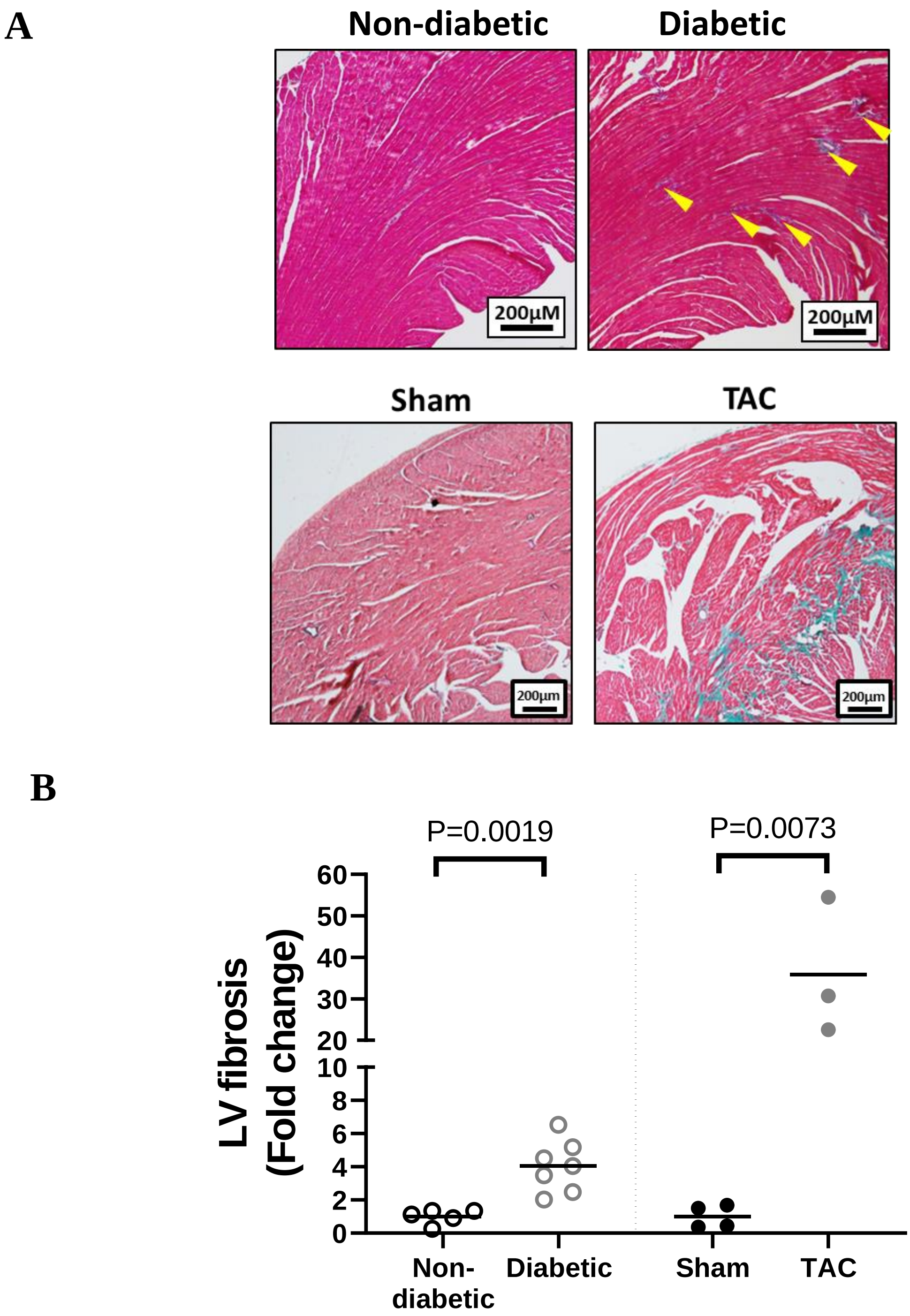

Figure S5: Fibrosis in the diabetic heart is modest in comparison to another model of cardiac disease – pressure overload due to transverse aortic constriction (TAC). (A) Representative cross-sections of non-diabetic and diabetic hearts from the current study (the same images as those presented in Fig 4A), and Sham and TAC hearts stained with Masson's trichrome. Scale bar = 200 µm. **(B)** Quantification of left ventricular (LV) fibrosis. Unpaired t-test. N=3-7 per group. Non-diabetic and diabetic values of LV fibrosis come from the current study but are expressed as a fold change (non-diabetic normalized to 1.0). Sham and TAC values of LV fibrosis come from a previous study (sham normalized to 1.0; Bernardo et al., PLOS ONE. 2014;9:e90337).

Table S1: microRNA primer sequences

Gene	TaqMan® Gene Expression AssayID	Mature miRNA/ Control sequence (5’-3’)	Dye	RefSeq
mmu-miR-34a-5p	000426	UGGCAGUGUCUUAGCUGGUUGU	FAM	MIMAT0000542
mmu-miR-34b-5p	002617	AGGCAGUGUAAUUAGCUGAUUGU	FAM	MIMAT0000382
mmu-miR-34c	000428	AGGCAGUGUAGUUAGCUGAUUGC	FAM	MI0000743
mmu-miR-92a	000430	UAUUGCACUUGUCCCGGCCUG	FAM	MIMAT0000539
Sno-U6	001973	GTGCTCGCTTCGGCAGCACATATACTAAAATTGGAACGATACAGAGAAGATTA GCATGGCCCCTGCGCAAGGATGACACGCAAATTCGTGAAGCGTTCCATATTTT	FAM	NR004394

Table S2: mRNA primer details - Taqman

Gene	TaqMan® Gene Expression Assay	Amplicon Length (bp)	Exon Spanning	Dye	RefSeq
natriuretic peptide type A; ANP *(Nppa)*	Mm01255747_g1	85	Yes	FAM	NM_008725.2
natriuretic peptide type B; BNP *(Nppb)*	Mm01255770_g1	68	Yes	FAM	NM_001287348.1<br>NM_008726.5
ATPase, Ca++ transporting, cardiac muscle, slow twitch 2; SERCA2a *(Atp2a2)*	Mm01201431_m1	90	Yes	FAM	NM_001110140.3<br>NM_009722.3<br>NR_027838.1
B cell leukemia/lymphoma 2 (*Bcl2*)	Mm00477631_m1	85	Yes	FAM	NM_009741.4
B cell leukemia/lymphoma 6 (Bcl6)	Mm00477633_m1	112	Yes	FAM	NM_009744.3
collagen, type I, alpha 1; Collagen 1 *(Col1a1)*	Mm00801666_g1	89	Yes	FAM	NM_007742.3
collagen, type III, alpha 1; Collagen 3 *(Col3a1)*	Mm00802300_m1	88	Yes	FAM	NM_009930.2
Connective tissue growth factor (*Ctgf*)	Mm01192932_g1	61	Yes	FAM	NM_010217.2
Cyclin D1 (Ccnd1)	Mm00432359_m1	58	Yes	FAM	NM_007631.2
Hypoxanthine guanine phosphoriboxyl transferase; *Hprt1*	Mm01545399_m1	81	Yes	VIC	NM_013556.2
myosin, heavy polypeptide 6, cardiac muscle, alpha; αMHC *(Myh6)*	Mm00440359_m1	67	Yes	FAM	NM_001164171.1<br>NM_010856.4
myosin, heavy polypeptide 7, cardiac muscle, beta; βMHC *(Mhy7)*	Mm01319006_g1	66	Yes	FAM	NM_080728.2

Gene	TaqMan® Gene Expression Assay	Amplicon Length (bp)	Exon Spanning	Dye	RefSeq
Neurogenic locus notch homolog protein 1 (Notch 1)	Mm00435249_m1	76	Yes	FAM	NM_008714.3
Cytochrome b-245, NADPH oxidase 2; (*Nox2*)	Custom designed probe			FAM	**Probe Sequence (6-FAM 5'-3'):**<br>CAACTGGACAGGAACCT<br>**Forward Primer:**<br>AGTGCGTGTTGCTCGACAAG<br>**Reverse Primer:**<br>CCAAGCTACCATCTTATGGAAAGT
Protein O-Fucosyltransferase 1 (*Pofut1*)	Mm00475567_m1	64	Yes	FAM	NM_080463.3
peroxisome proliferative activated receptor, gamma, coactivator 1 alpha; PGC1α *(Ppargc1a)*	Mm01208835_m1	68	Yes	FAM	NM_008904.2<br>NR_027710.1
Protein Phosphatase 1 Regulatory Subunit 1, Ppp1r10; *Pnuts*	Mm01271041_m1	61	Yes	FAM	NM_001163818.1
Neutrophil cytosol factor 1**,** p47phox; *p47*	Custom designed probe			FAM	**Probe Sequence (6-FAM 5'-3'):**<br>CCCAGCCTTCTGCAGAT<br>**Forward Primer:**<br>CCGGCTATTTCCCATCCAT<br>**Reverse Primer:**<br>TCGCTGGGCCTGGGTTAT
Semaphorin-4B (Sema4b)	Mm00803797_m1	69	Yes	FAM	NM_013659.4
Sirtuin 1 (*Sirt1*)	Mm00490758_m1	96	Yes	FAM	NM_001159589.1<br>NM_019812.2
Tumor Necrosis Factor Alpha (*Tnfa*)	Mm00443260_g1	61	Yes	FAM	NM_013693.3

Gene	TaqMan® Gene Expression Assay	Amplicon Length (bp)	Exon Spanning	Dye	RefSeq
Vascular Endothelial Growth Factor A (*Vegfa*)	Mm01281449_m1	81	Yes	FAM	NM_001025250.3<br>NM_001110266.1<br>NM_001110267.1<br>NM_001287056.1<br>NM_001287057.1<br>NM_009505.4
Vascular Endothelial Growth Factor B (*VegfB*)	Mm00442102_m1	93	Yes	FAM	NM_001185164.1<br>NM_011697.3
Vinculin (*Vcl*)	Mm00447745_m1	70	Yes	FAM	NM_009502.4

Table S3: mRNA primer details - Sybr

Gene	Forward primer	Reverse Primer	Amplicon length	Single peak in Melt Curve
TIMP metallopeptidase inhibitor 1 (TIMP1)	CAGATACCATGATGGCCCCC	CGCTGGTATAAGGTGGTCTCG	190	Yes
TIMP metallopeptidase inhibitor 2 (TIMP2)	CCAGAAGAAGAGCCTGAACCA	GTCCATCCAGAGGCACTCATC	112	Yes
TIMP metallopeptidase inhibitor 3 (TIMP3)	GGCCTCAATTACCGCTACCA	CTGATAGCCAGGGTACCCAAAA	135	Yes
TIMP metallopeptidase inhibitor 4 (TIMP4)	TGCAGAGGGAGAGCCTGAA	GGTACATGGCACTGCATAGCA	80	Yes
Matrix metalloproteinase-2 (MMP2)	CACACCAACACTGGGACCTG	AGAATGTGGCCACCAGCAAG	167	Yes

Table S4: Morphological and systemic characteristics of non-diabetic and diabetic mice treated with saline or LNA antimiR-34a after 16 weeks of diabetes.

	**Non-diabetic**		**Diabetic**			
	**Saline**		**Saline**		**LNA antimiR-34a**	
**No. of animals**	11		11		13	
**Age (weeks)**	23.2	± 0.2	23.1	± 0.2	23.2	± 0.2
**Blood glucose (mmol/L, endpoint) ^**	17.9	± 0.8	33.3	± 0.0*	33.2	± 0.1*
**HbA1c (NGSP %)**	4.0	± 0.0	9.2	± 0.4*	9.8	± 0.4*
**HbA1c (IFCC mmol/mol)**	20.2	± 0.2	76.5	± 4.1*	83.6	± 3.9*
**Body weight (g)**	29.0	± 1.0	31.0	± 0.6	30.1	± 0.8
**Tibial length (mm)**	16.3	± 0.1	16.6	± 0.1	16.4	± 0.1
**Heart weight (mg)**	124.1	± 2.3	133.0	± 2.3#	128.4	± 3.3
**Atrial weight (mg)**	9.1	± 0.4	10.9	± 0.4*	11.1	± 0.4*
**Lung weight (mg)**	144.7	± 3.7	153.4	± 2.3	158.3	± 6.0
**Liver weight (mg)**	1187.9	± 34.9	1903.9	± 59.5*	1925.4	± 38.8*
**Kidney weight (mg)**	417.4	± 12.6	659.2	± 42.6*	656.6	± 43.3*
**Spleen weight (mg)**	80.7	± 2.1	98.9	± 5.3*	95.1	± 4.3*
**HW/BW (mg/g)**	4.3	± 0.1	4.3	± 0.1	4.3	± 0.1
**AW/BW (mg/g)**	0.32	± 0.02	0.35	± 0.01	0.37	± 0.02
**LW/BW (mg/g)**	5.0	± 0.1	5.0	± 0.1	5.3	± 0.2
**LivW/BW (mg/g)**	41.1	± 0.6	61.6	± 1.7*	64.5	± 1.9*
**KW/BW (mg/g)**	14.5	± 0.4	21.4	± 1.6*	22.3	± 2.2*
**SW/BW (mg/g)**	2.8	± 0.1	3.2	± 0.2	3.2	± 0.2
**HW/TL (mg/mm)**	7.6	± 0.1	8.0	± 0.1#	7.8	± 0.2
**AW/TL (mg/mm)**	0.56	± 0.03	0.66	± 0.02*	0.68	± 0.03*
**LW/TL (mg/mm)**	8.9	± 0.2	9.3	± 0.1	9.6	± 0.3
**LivW/TL (mg/mm)**	72.8	± 1.9	114.9	± 3.5*	117.3	± 2.2*
**KW/TL (mg/mm)**	25.6	± 0.7	39.8	± 2.6*	40.0	± 2.8*
**SW/TL (mg/mm)**	4.9	± 0.1	6.0	± 0.3*	5.8	± 0.3*

Abbreviations: AW, atria weight; BW, body weight; HbA1c, hemoglobin A1c; HW, heart weight; IFCC, International Federation of Clinical Chemistry and Laboratory Medicine; KW, kidney weight; LW, lung weight; LivW, liver weight; NGSP, National Glycohemoglobin Standardization Program; SW, spleen weight; TL, tibial length. Data are shown as mean±SEM. Data analyzed using a one way ANOVA with Fisher's post-hoc Test, except for blood glucose and HbA1c: data analyzed using a Kruskal-Wallis non-parametric one way ANOVA with Dunn's Post Hoc. *P<0.05 vs. non-diabetic mice. #P<0.05 by unpaired t-test vs non-diabetic

mice. ^Note: N=8 (Non-diabetic, diabetic saline), 10 (diabetic LNA antimiR-34a) for blood glucose at endpoint. Not assessed on 3 mice at 4, 8, 12, & 16 wks for animal welfare reasons, but diabetic based on 2 week reading and endpoint HbA1c.

Table S5: Pulsed waved and tissue Doppler echocardiography data in non-diabetic and diabetic mice.

	BASELINE			8 WEEKS POST-STZ (before treatment)			ENDPOINT (after treatment)		
	Non-diabetic	Diabetic		Non-diabetic	Diabetic		Non-diabetic	Diabetic	
	Saline	Saline	LNA antimiR-34a	Saline	Saline	LNA antimiR-34a	Saline	Saline	LNA antimiR-34a
No. of animals	9	11	10	9	11	10	9	11	10
Body weight (g)	22.2±0.7	24.4±0.4*	24.2±0.5*	28.1±0.7^	29.6±0.4^	29.1±0.6^	30.3±1.0^†	31.2±0.4^†	30.2±1.1^
Pulsed Wave Doppler									
Heart rate (bpm)	384±7	391±8	392±5	367±8	394±7*	397±8*	382±9	394±7	418±11*^
E wave (mm/s)	762.6±25.5	707.0±18.0	730.8±20.3	740.8±36.9	730.6±23.8	720.2±31.2	740.2±34.1	692.6±29.3	721.0±39.9
A wave (mm/s)	381.1±22.8	377.6±12.0	363.6±11.8	343.9±16.1	412.7±12.8*	419.9±19.7*	332.2±15.7	415.5±27.4*	409.4±38.6
E/A ratio	2.1±0.1	1.9±0.1	2.1±0.1	2.2±0.2	1.8±0.1*	1.7±0.1*^	2.3±0.1	1.7±0.1*	1.9±0.1*
Tissue Doppler									
Heart rate (bpm)	381±6	393±7	389±5	365±8	393±7*	396±7*	378±8	392±8	407±9*
e' (mm/s)	-23.8±0.8	-23.6±1.1	-23.1±1.0	-24.9±1.8	-22.5±1.2	-23.0±1.2	-26.2±1.8	-22.2±1.9	-22.6±1.7
a' (mm/s)	-15.5±0.7	-17.6±0.9	-16.4±0.8	-13.9±0.9	-16.5±0.6*	-17.9±0.6*	-14.1±0.6	-18.1±1.4*	-18.1±1.5*
e'/a' ratio	1.6±0.1	1.4±0.1	1.4±0.1	1.9±0.2	1.4±0.1	1.3±0.1*	1.9±0.1	1.3±0.1*	1.3±0.1*
E/e' Ratio	32.2±1.3	30.7±1.8	32.0±1.2	30.8±2.3	33.0±1.4	31.8±1.3	29.0±1.8	33.9±3.7	33.2±2.6

Abbreviations: A wave, peak late mitral inflow wave velocity; a', peak late mitral annular tissue velocity; E wave, peak early mitral inflow wave velocity; e', peak early mitral annular tissue velocity; STZ, streptozotocin; Data are shown as mean±SEM. Data analyzed by two way repeated measures ANOVA with Fisher's post-hoc test. *P<0.05 *vs.* Non-diabetic at same time point, ^P<0.05 *vs.* baseline of same group, †P<0.05 *vs.* 8 weeks of same group.
